# Monitor displays in radiology: Part 1

**DOI:** 10.4103/0971-3026.45341

**Published:** 2009-02

**Authors:** IK Indrajit, BS Verma

**Affiliations:** Department of Radiodiagnosis and Imaging, Army Hospital (Research and Referral), Delhi Cantt – 110 010, India; 1 Department of Radiodiagnosis and Imaging, Base Hospital, Lucknow – 226 002, India

**Keywords:** CRT/ LCD display, monitor displays, passive and active LCD matrix, flat panel monitor display

## Abstract

Monitor displays are an integral part of today's radiology work environment, attached to workstations, USG, CT/MRI consoles and PACS terminals. For each modality and method of use, the correct display monitor needs to be deployed. It helps to have a basic understanding of how monitors work and what are the issues involved in their selection.

## Introduction

Monitor displays are commonly used peripheral output devices in computers. These peripheral devices are also called ‘display monitors’ or ‘monitors’ or ‘displays’. They display information to a computer user.[1] There are a few important reasons why practicing radiologists should have a working knowledge of monitor displays and these are described below.

**Impact of digital imaging:** Computers play an important role in contemporary radiology practice. Most radiology modalities today use monitor displays to aid analysis of images. Monitors have become integral components of digital radiography, USG, CT / MRI consoles and workstations, and PACS terminals.

**Image chain:** There is an image chain that radiologists need to be aware of while working on computers with monitor displays. At one end of the image chain is the modality. Here pixels, gray scale values, processing, postprocessing, and window level and width are important parameters that govern the appearance of any given image. In the middle of the image chain is the computer with its display controller, graphic cards, and look-up tables (LUT) memory, which influence the digital generation of an image. The human observer's visual system is the final element of the image chain. Its performance is strongly affected by ambient light, environment, reflection, veiling glare, angular response, and visual acuity.

**Shift in analysis model:** In the traditional model of radiology practice, hardcopy images displayed on viewboxes were the first point of analysis. Today, in most instances, softcopy images displayed on monitors are the first point of analysis. As a result, key steps like viewing, analysis, processing, and postprocessing of softcopy images are executed directly at monitors of consoles, workstations, and office desktops.[2]

**Heterogeneity of data:** The data displayed on the monitors in a radiology department is heterogeneous. It is often a variable combination of monochrome and gray-scale and/or color images viewed alongside text, audio, and/or video.[3] In such circumstances, radiologists need to possess a working knowledge of important performance parameters like resolution, brightness, contrast ratio, and viewing angles.

**Growth of RIS, PACS, and teleradiology:** Image transfer across a variety of networks and radiology modalities is common practice these days. Images are increasingly being stored as part of a patient's electronic medical records, to be analyzed as and when required; images are often transferred over departmental networks and to teleradiology workstations for analysis[3] In such a diverse set of locations, it is common to find different types of monitors used for displaying assorted types of data.

**Original dataset:** The American College of Radiology (ACR) has devised guidelines for monitor displays, based on the matrix size of the original digital image dataset. Monitors for *small matrix* datasets [typically sourced from CT, MRI, USG, nuclear medicine (NM), digital fluorography, and digital subtraction angiography (DSA)] have different performance guidelines as compared to monitors required for *large matrix* datasets [e.g., sourced from digital radiography (DR), computed radiography (CR), digitized films, and digital mammography][4]. The large matrix datasets require monitors with higher performance. As a rule of thumb, the resolution of the selected display system, ideally, should match the matrix of the image acquisition data.[4]

**Image consistency:** Each and every computer and its monitor at our workplace, handles gray-scale images in a different way. This is governed by factors such as acquisition parameters, application technique, graphics board, video board memory and processing, LUTs, and display signal processing. Therefore, there is a growing awareness of the need to maintain image consistency and gray-scale calibration across a broad variety of monitor displays.[5]

## Types of monitor display

The currently available medical monitors are classified by the American Association of Physicists in Medicine (AAPM) into primary and secondary display systems. Primary display systems are used for interpretation of medical images, as in radiology. They have to meet strict performance criteria. On the other hand, secondary display systems are used by staff other than radiologists, usually after an interpretative report has been rendered.[6]

**Figure 1:**
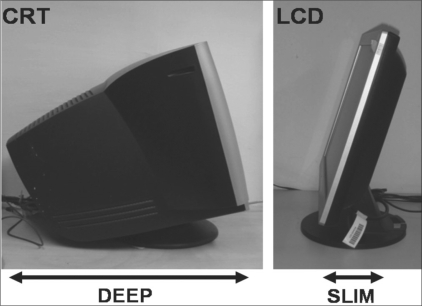
Profile view comparing CRT and LCD monitor displays. LCD monitor displays have the advantages of having smaller footprints and being perfectly flat, less heavy, thinner, and more adjustable

**Figure 2:**
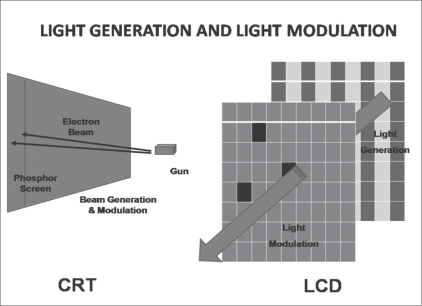
Functional differences between CRT and LCD displays. In CRT displays, light generation and light modulation are a single process, controlled by a single physical component. In LCD displays, light generation is physically separated from light modulation. Light generation (called backlight) is continuously on at full intensity. Light modulation shuts off light flux selectively by means of an LCD panel.

Over the years, two different monitor display technologies have emerged in the computer industry, i.e., the cathode ray tube (CRT) and the liquid crystal display (LCD). CRT is a mature technology, while the LCD is a recent innovation [Table 1]. LCD monitor displays have the advantages of having a smaller footprint and of being perfectly flat, less heavy, thinner, and more adjustable [Figure 1]. One significant difference between the two is in the integration of the key steps of light generation and modulation. In LCD monitors, light generation and light modulation are physically separated, unlike in CRT monitors [Figure 2].[7] Currently, LCD monitors are preferred over CRT displays.[4]

**Table 1:** Salient differences between cathode ray tube and liquid crystal display monitor display[8]

Parameters	CRT	LCD
Size and weight	Bulky and heavy	Slim and light weight
Technology	Mature	New
Response	Instantaneous speed	Poor speed response
Flicker	Image flicker present	No image flicker
Perfect black	Perfect black possible	Imperfect black
Sharpness	Less uniform sharpness	Uniform sharpness
Contrast ratio	Independent of viewing angle	Dependent on viewing angle
Image retention	No image retention	Image retention present
Aging	Due to phosphor	Due to backlight aging
Power consumption	High	Low

CRT: Cathode ray tube, LCD: Liquid crystal display

## CRT monitor display technology

The CRT was invented by Karl Ferdinand Braun, a German physicist in 1897.[9,10] Simply put, it is an electronic vacuum tube that features a focused beam of electrons.[10] CRT monitor displays comprise an electron-generating assembly and a display screen.[1] The electron-generating assembly consists of a *cathode ray tube*, *electron guns*(traditionally three in number in color monitors),[11] *focusing / deflection coils* that modulate the beam, and a *mask* for selectively blocking/allowing red, green, or blue beams before displaying an image. The display screen is coated with a phosphor layer having millions of tiny red, green, and blue phosphor dots.

In CRT monitors, the cathode is a heated filament within a vacuum created inside a glass tube. The emanating electrons are negatively charged while the screen has a positive charge. The electron beam travels across the vaccum tube at high speeds, striking the display screen from left to right and from top to bottom. The phosphor dots in the display screen glow when struck by the electron beam, thereby creating a visible image.

There are a few important differences between black and white (monochrome) and color CRT monitors. A monochrome CRT monitor has one beam, whereas color CRT monitors have three electron beams (red, green, and blue) that sweep simultaneously across the display screen while forming an image. The display screen has three coats of phosphors (red, green, and blue) arranged in dots or stripes. Color CRT monitors, in addition, have a shadow mask, which is a perforated, thin metal screen having holes that are aligned with the phosphor dots. Table 2 lists the advantages and limitations of CRT monitor displays.

**Table 2:** Advantages and limitations of cathode ray tube monitors

Advantages	Limitations
• Better color clarity and depth[12]	• Bulky, heavy, and uses space on desk[15]
• High refresh rates[12]	• Constant refreshing can result in headache[15]
• High contrast[1]	• Operates at very high voltage; overheats system[15]
• More responsive[13]	• Strong vacuum may result in implosion^[115]^
• Less ghosting and blurring[13]	• Health hazard due to electromagnetic field emission[15]
• Flexible, with multiple resolutions[14]; multisync capable[12]	• Limited brightness level[14]
• Image quality superior to that with LCD/plasma	• Decreased brightness and sharpness at edges[16]
• Lower cost than LCD or plasma[15]	• Phosphor efficiency fades with time[16]
	• Phosphor burn artifacts, with ‘ghost negative images[1,16]
	• Impedance mismatch artifacts, with ‘bleeding’ of white into black and vice versa at black /white interfaces[16]

## LCD monitor technology

### Basis of LCD

A liquid crystal is a rod-shaped substance ‘that flows like a liquid but maintains some of the ordered structure characteristic of crystals.’[17,18] It was discovered in 1888 by an Austrian botanist, Fredreich Rheinizer.

A naturally twisted type of liquid crystal is called twisted nematics (TN). It is the basic ingredient of the LCD technology prevailing today. It is cost-effective due to its ease of manufacture and its simplicity of structure. When subject to an electric current, it ‘untwists to varying degrees, depending on the current's voltage.’[10] Furthermore, TN has the unique ability to rapidly twist and untwist. This therefore allows a quick response while switching to and fro between dark and bright states and vice versa.

Under normal conditions, light is a collection of electromagnetic waves that vibrate in all directions. Polarization is a distinctive phenomenon wherein the light wave vibrations move in a single plane.[19] This is achieved by sending light through a polarizing filter, which selectively ensures that light vibrates in one direction while blocking all light that vibrates in other directions.

LCD technology is practical due to the following factors: a) light can be polarized, b) liquid crystals can transmit and change polarized light, c) the structure of liquid crystals can be changed by an electric current, and d) there are transparent substances that can conduct electricity.[10]

### Components of an LCD monitor display

**Figure 3:**
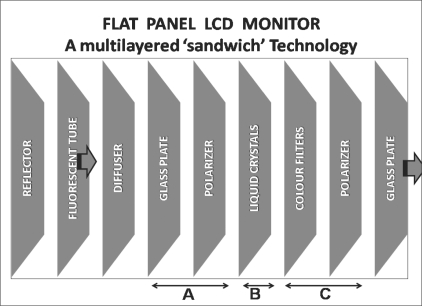
Flat panel LCD monitor technology with a multilayered ‘sandwich’ configuration, comprising of LCD panel and backlight. a) A TFT glass polarizer has TFTs proportional to the number of pixels displayed. b) Liquid crystals shift according to the difference in voltage between the color filter glass and the TFT. c) A color filter glass generates color.

An LCD monitor is fundamentally a multilayered light valve,[7] a ‘sandwich’[15] comprising three main components: an LCD panel, a backlight, and an inverter[10] [Figure 3].

An *LCD panel* has two pieces of polarized glass substrate within which thousands of liquid crystal pixels are arranged in tiny containers called cells. The crystals form the tiny pixels seen as colors during the creation of an image on the screen.[15] A *backlight* is a fluorescent light source that passes through the first substrate. Electrical currents around the edge of the LCD twist the liquid crystal molecules[15] to align them and allow varying levels of light to pass through to the second substrate, which creates the colors and images. The backlight is continuously on at full intensity and, eventually, ‘only 5-10% of the original backlight remains as the light reaches the front side of an LCD screen.’[20] *Inverters* send power to the backlights.

### Passive and active LCD matrix

LCD panels with a passive matrix use a grid of conductive metal to charge each pixel. Though less expensive to produce, these are rarely used today because of ‘slow response time and imprecise voltage control.’[21]

In comparison, active matrix LCDs are made of tiny transistors and capacitors on the glass of the display. Thin-film transistor (TFT) LCD technology ensures that individual pixels are addressed in rows and columns, which reduces the connection count from millions to thousands.[10] However, a large amount of power is required for the numerous transistors. Currently, most LCD displays are TFT-based, using the active matrix technology.[1]

## Flat-panel monitor display

A flat-panel monitor design has inherent advantages. It eliminates distortion artifacts, has a lower susceptibility to light reflections, is shallower, and is light in weight. Most flat-panel displays use LCD technology,[11] which is conducive to the manufacture of thin, perfectly flat, light-weight, and adjustable displays. Unfortunately, when compared with curved-surface monitors, flat-panel monitors are more dependent on a rectangular viewing angle to achieve optimal performance.[22]

## Advantages and limitations of LCD monitor display

With the increasing use of LCD technology in medical displays, it has been observed that LCD technology is less susceptible to failures than CRT technology due to reasons such as lower voltage use, lower power consumption, and lower maintenance cost.[8] The advantages and limitations of LCD monitor displays are listed in Table 3.

**Table 3:** Advantages and limitations of liquid crystal display monitor display

Advantages	Limitations
• Flat, with space-saving design[15]	• Slow response times with limited refresh rates[14]
• Small footprint	• Fixed or ‘native’ resolution display[1]
• Thinner, weighs less[1,12]	• Limited viewing angles[15]
• More adjustable[13]	• Unsatisfactory video quality[12,23]
• Consumes little electricity[13,15]	• Poor black level: best black is an extreme dark gray
• Produces little heat[15]	• Lifespan limited by backlight life
• High peak intensity produces bright images[15]	• Higher cost
• Lack of flicker and low glare reduces eyestrain[12,13]	• Some pixels die, leaving a discolored black spot on the display[1,15]
• Suited ideally for brightly lit environments[15]	• ‘Screen door’ effect due to transistors and signal wires running between pixels[14]

The details of the various performance parameters for monitor displays and quality issues related to medical grade displays will be covered in Part II of this article.
